# swDMR: A Sliding Window Approach to Identify Differentially Methylated Regions Based on Whole Genome Bisulfite Sequencing

**DOI:** 10.1371/journal.pone.0132866

**Published:** 2015-07-15

**Authors:** Zhen Wang, Xianfeng Li, Yi Jiang, Qianzhi Shao, Qi Liu, BingYu Chen, Dongsheng Huang

**Affiliations:** 1 Research Center of Blood Transfusion Medicine, Key Laboratory of Laboratory Medicine (Wenzhou Medical University), Ministry of Education, Zhejiang Provincial People's Hospital, Hangzhou, Zhejiang, China; 2 State Key Laboratory of Medical Genetics, Central South University, Changsha, Hunan, China; 3 Institute of Genomic Medicine, Wenzhou Medical University, Wenzhou, Zhejiang, China; NIDCR/NIH, UNITED STATES

## Abstract

DNA methylation is a widespread epigenetic modification that plays an essential role in gene expression through transcriptional regulation and chromatin remodeling. The emergence of whole genome bisulfite sequencing (WGBS) represents an important milestone in the detection of DNA methylation. Characterization of differential methylated regions (DMRs) is fundamental as well for further functional analysis. In this study, we present swDMR (http://sourceforge.net/projects/swdmr/) for the comprehensive analysis of DMRs from whole genome methylation profiles by a sliding window approach. It is an integrated tool designed for WGBS data, which not only implements accessible statistical methods to perform hypothesis test adapted to two or more samples without replicates, but false discovery rate was also controlled by multiple test correction. Downstream analysis tools were also provided, including cluster, annotation and visualization modules. In summary, based on WGBS data, swDMR can produce abundant information of differential methylated regions. As a convenient and flexible tool, we believe swDMR will bring us closer to unveil the potential functional regions involved in epigenetic regulation.

## Introduction

DNA methylation, catalyzed by DNA methyltransferases (DNMTs), occurs primarily on carbon 5 position of cytosine bases and plays a pivotal role in transcriptional regulation, chromosome stability, genomic imprinting, X-inactivation and tissue differentiation[1–5]. Evidence suggests that regions of methylated DNA are correlated with the expression of several tissue-specific genes[4] and shown influences on activating coding regions across the genome[6, 7]. Aberrant DNA methylation was reported to be implicated in the etiology of various diseases and may promote the development of cancer[8, 9]. Therefore, identification of genomic regions with differential methylation level, termed as differentially methylated regions (DMRs), represents the most important and fundamental step in dissecting these functional regions that may be involved in transcriptional regulation.

Recently, the advent of the whole genome bisulfite sequencing (WGBS) has made a stride in the progress of DNA methylation analysis at single-base resolution[10–14]. This high-throughput technology has been applied to quantitative measurement of whole genome DNA methylation (methylome). Subsequently, precision DMRs could be identified from this single-base resolution methylome. Conventional practice uses bisulfite treatment deaminating unmethylated cytosines to uracil, which is later converted into thymine in DNA, making DNA sequence contain only A, T and G. High-throughput sequencing generates ternary reads of lower complexity, followed by algorithmic tools to align reads to reference and statistical analysis to discern cytosine and methylated cytosine. Many previous studies working on identifying DMRs from WGBS data have been successfully implemented, which provided novel insights about genomic placement and functional consequences of DNA methylation in cancer[8, 9].

To date, a number of tools have been developed and available for DMRs identification from methylomes. For example, dmrFinder[15], QDMR[16] and methylMnM[17] are designed for the analysis of DMR based on microarray, MeDIP-seq or MRE-seq data. To perform methylome DMR detection at single-base resolution, CpG_MPs[18], DSS[19], bsseq[20], eDMR[21], methylSig[22], MOABS[23], ComMet[24] and BiSeq[25] adopt different strategies (Table 1). Most of them require samples with two more replicates except CpG_MPs, ComMet and BiSeq. Despite the fact that proper replicates are essential to reduce bias from individual sequencing data, the high cost of WGBS diminished the feasibility of these approach, thus identifying DMRs from methylome without replicates receives more popularity.

**Table 1:** Software of DMR detection.

Software	Method	Samples	Replicates
DSS	Dispersion shrinkage estimate Gamma-Poisson or Beta-Binomial distributions	two groups	yes
bsseq	Smoothing t-test	two groups	yes
methylSig	Beta-binomial	two groups	yes
eDMR	Expectation maximization (EM) algorithm to fit to a bimodal normal distribution	two groups	yes
MOABS	Beta-Binomial hierarchical model	two groups	yes
BiSeq	Design for targeted BS data (RRBS)	two or groups	no
CpG_MPs	Combined hotspot and entropy theory	two or more	no
ComMet	Use HMM model to identify DMR (a tool of bisulfighter).	two	no

To facilitate the identification of DMRs from those methylomes without replicates, we developed swDMR which integrates multiple statistical methods based on a sliding window approach to suffice easy detection, annotation and visualization of DMRs from WGBS across multiple samples.

## Implementation

### Data input

**Fig 1:**
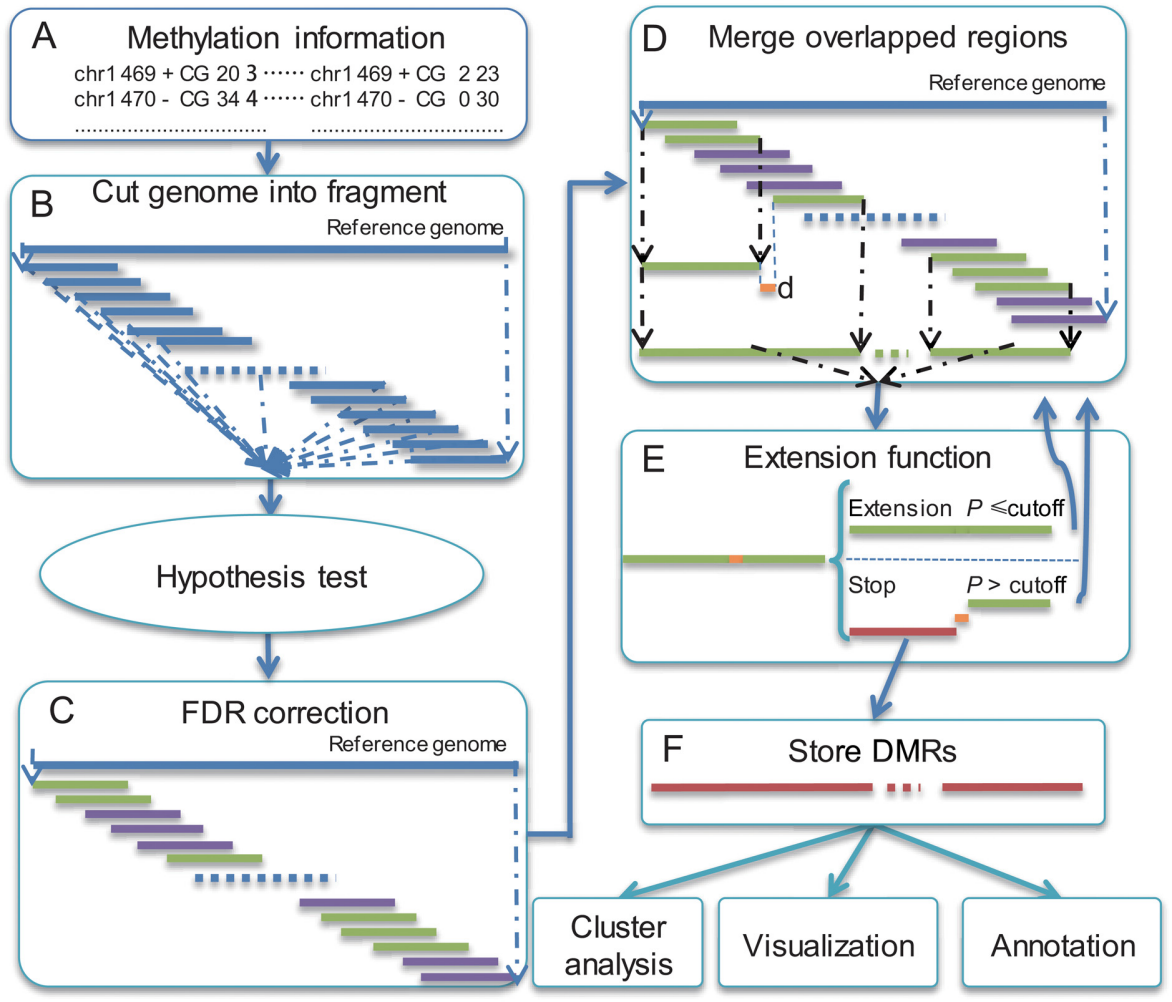
Workflow of swDMR. (A) Prepare methylation information of cytosines for swDMR. (B) Cut genome into fragments with defined window and step size. (C) Adjust *P* value with FDR correction method. (D) Merge overlapped potential DMR regions. (E) Filter out regions with larger *P* value than cutoff. Blue fragments represent overlapped region; green fragments represent potential DMR; pink fragments represent non-potential DMR; Red regions represent DMRs.

swDMR requires input files containing basic information of methylation cytosine across multiple samples, including chromosome numbers, genomic coordinates, type of cytosine (CG, CHG and CHH), numbers of methlylated cytosine (C) and unmethylated cytosine (T) (Fig 1A). Various WGBS data aligners[26], such as Bismark, BRAT, BS-Seeker, MethyCoder, SOCS-B and B-SOLANA, were not integrated in swDMR, but they can be selected for users to easily align reads through WGBS to the reference genome and generate methylation information of each cytosine. To make swDMR more convenient, we recommend user to use Bismark to prepare input data of swDMR, given that the output of Bismark could be used to swDMR directly.

### DMR detection and annotation

Once the input file has been determined, the following procedures will be applied to detect DMR (Fig 1B–1F).

Firstly, with a sliding window algorithm based on defined window size and step size, the whole genome are divided into multiple fragments with overlapping regions of equal length. Those sliding windows, which are used for further statistical analysis, should meet the three criteria as follows: a) the depth in each cytosine position should be more than defined threshold in each sample. The filteration is based on the depth of each cytosine which is defined by user. It is flexible that users can adjust the thresholds based on the sequencing depth freely. Meantime, we provide default thresholds (default is 4) for 30X data; b) number of selected cytosine, remaining through previous condition, should be larger than defined value; c) after calculating mean methylation level of each sample, the fold changes and differences of mean methylation level between the two samples with maximum and minimum methylation level should be larger than defined values, respectively. Secondly, choose one suitable statistical method to perform hypothesis test. swDMR has integrated several commonly used methods, containing both parametric and non-parametric methods (T-test, Wilcoxon, Chisquare, Fisher, ANOVA and Kruskal wallis test), adapted to DMR detection for multiple samples. Each sliding window will acquire one P value with the selected statistics test method. Thirdly, false discovery rate (FDR) method, proposed by Benjamini and Hochberg[27] to corrected P value, will be implemented. Those regions with adjusted P value less than the cutoff of FDR are defined as potential DMRs. Then, we proposed an extension function. Through the extension function, we merged two potential DMRs if the distance between the two potential DMRs was less than the threshold user-defined. The merged potential DMRs would be subject to statistical test previously selected to guarantee that the merged region is significantly different. The current extension step will stop until the re-performed P value exceeds the pre-defined cutoff. Then a new extension of the left potential DMRs will start. After repeating extension steps, those merged regions with P value less than user-defined cutoff are then defined as candidate DMRs. Lastly, the DMR length distribution will be calculated. To obtain global trends of DNA methylomes in DMRs among multiple samples, swDMR perform complete linkage hierarchical clustering of the methylation level to the corresponding samples, using the heatmap.2 R function in gplots package. BEDTools[28] is implemented for flexible annotation of DMRs by comparing the chromosome coordinate of DMRs with the corresponding annotation information in GFF/GTF/BED format. To reveal the overview of DNA methylation patterns for one specific DMR, the methylation information of DMRs with up- and down-stream flanking regions were plotted. swDMR also provides WIG format files to query against UCSC genome browser [29], in which functional genomic elements of relevant DMRs can be visualized optionally. Alternatively, WIG format file can be also visualized on IGV[30] with self-defined elements.

### Comparison with other DMR detection methods

Some existing tools, such as CpG_MPs, ComMet and BiSeq have been applied to DMR detection without replicates. BiSeq, which is mainly designed for analyzing targeted bisulfite sequencing data, may not make a good performance for WGBS; while CpG_MPs, which combined hotspot algorithm and entropy, can be applied to DMR detection without replicates. ComMet, a tool packaged in Bisulfighter[24], employed HMM model to detect DMR without replicates. Finally, to confirm the practical utility of swDMR to samples without replicates, CpG_MPs and ComMet were implemented to do the comparison with swDMR.

### Simulation data

**Fig 2:**
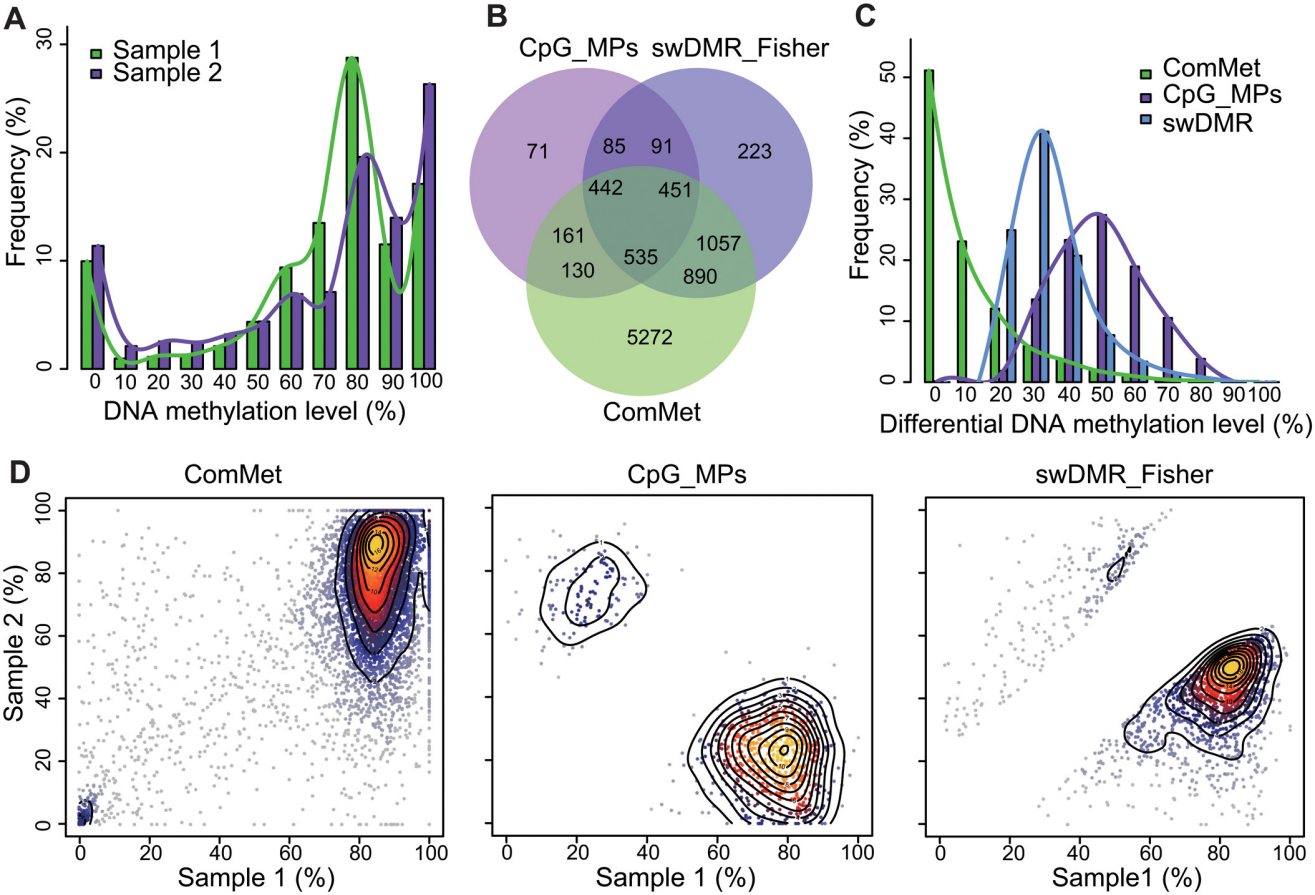
Simulation data and DMR detection comparison. (A) Simulation DNA methylation level distribution of two samples. (C) Distribution of absolute differential methylation level in DMR. (C) Venn diagram of ComMet (lightgreen), CpG_MPs (darkorchid) and swDMR (cornflowerblue). (D) Scatter plot of DNA methylation level in DMRs of simulation data to three DMR detection methods. Color (ranged from gray to yellow with contour line) represents intensity of DMRs.

We took chromosome 21 to simulate two methylome data sets of bisulfite sequencing with bimodal distribution (Fig 2A).

DMR detection was applied with CpG_MPs, ComMet and swDMR respectively. By default, a window size of 1000bp and a step size of 100bp were defined. The coverage of CpGs in defined regions should be at least 5X and the fold change of methylation level should be at least 1.5 with more than 0.2 differences between compared samples. The number of CpGs in those regions is required at least 5, the P value and FDR value of candidate DMRs by fisher exact test should be less than 0.01. To implement CpG_MPs, we defined more than 5 CpGs in the hotspot region. Then, CpG_MPs and ComMet were applied using the above parameters.

CpG_MPs, ComMet and swDMR identified 759, 6,827 and 1,822 DMRs respectively (Fig 2B). We found that swDMR identified more DMRs with absolute methylation level difference ranging from 20% to 40% while CpG_MPs preferred to identify DMRs with larger absolute difference of methylation level (Fig 2C). However, ComMet identified 74.21% DMRs with absolute differential methylation level less than 10% (Fig 2C). Compared with CpG_MPs and swDMR, ComMet identified the most regions in high or low methylation levels of two samples (Fig 2D), which indicated that those DMRs identified by ComMet with low absolute difference of methylation level would be false positive. In conclusion, with the additional manipulation of differential methylation level which is necessary for DMRs detection, swDMR find more DMRs than CpG_MPs.

### Real data

Considering the high potential false positive, ComMet was not implemented to the subsequent analysis.

To compare CpG_MPs and swDMR in real data set, we obtained single-base resolution methylome data of three cell lines (human embryonic stem cell (hESC), fibroblast-like cells differentiated from hESCs (hESC-Fibro) and primary neonatal foreskin fibroblasts (Fibro), respectively) from GEO database with accession GSE19418[12]. Previous study uncovered decrease of global DNA methylation level during the differentiation process from hESC to hESC-Fibro and from hESC-Fibro to Fibro. For DMRs detection with CpG_MPs, we used the same parameters as used in simulation data. Meanwhile, using swDMR with other same parameters, fisher exact test and ANOVA were applied to DMRs detection for two samples and three samples, respectively.

### Two samples

Methylome of hESC and Fibro were used for DMR detection.

**Fig 3:**
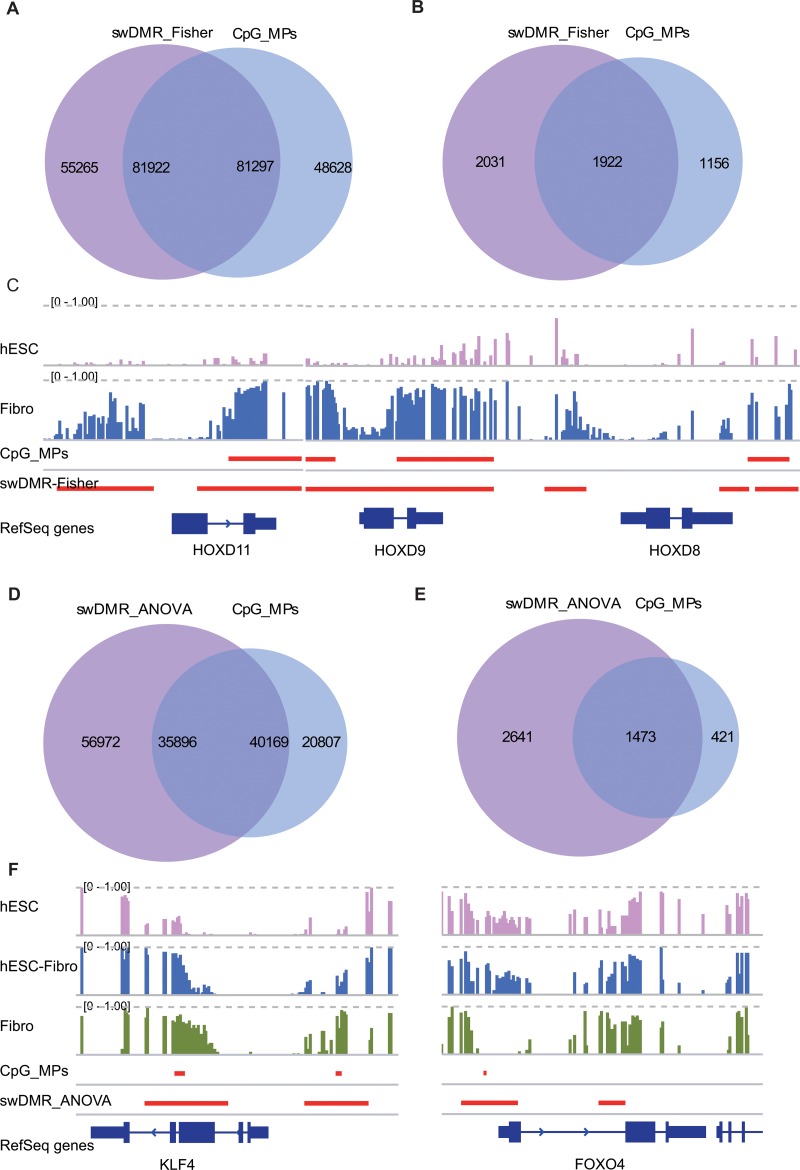
Comparison of swDMR and CpG_MPs in two and three samples. (A, D) Venn diagram of DMRs detected by fisher exact test through swDMR and CpG_MPs to two samples respectively. (B, E) Venn diagram of genes with DMRs overlapped in their promoters (promoter: -1.3kb of TSS and +0.2kb of TSS). (C, F) swDMR specific genes with methylation level of hESC, hESC-Fibro and Fibro. Methylation of each CpG ranges from 0 to 1. Red lines represent DMRs of swDMR and CpG_MPs. Blue track represents RefSeq genes.

After DMR detection process, swDMR and CpG_MPs identified 137,187 and 129,925 DMRs respectively. About 62.57% (81,297 in 129,925) DMRs identified by CpG_MPs have intersections with that of swDMR (Fig 3A). These DMRs were annotated to gene features (For example: UTR, CDS, Promoter, Upstream, Downstream, et al. S1 Table). Given the fact that promoters are generally with hypomethylation and aberrant DNA methylation in promoter plays vital role in gene expression, we focused on genes with DMRs overlapped with their promoters. In this comparison, there were 1,922 genes with DMRs in promotors jointly found by swDMR and CpG_MPs, while 1,156 and 2,013 genes were exclusively found by CpG_MPs and swDMR respectively (Fig 3B). Among 2,013 swDMR specific genes, several genes are related with hESC and Fibro development, such as HOXD11, HOXD9 and HOXD8 (Fig 3C). By GO enrichment analysis of those three part genes were processed by an R package GOstats, we found that those 1,922 shared genes were significantly enriched in 255 biology processes (P value < 0.01, S2 Table), including multicellular organismal process, tissue development, cell migration, anatomical structure development, system development, developmental process, stem cell differentiation et al. These processes are closely related to hESC developing to Fibro. Also, up to 1,156 specific genes of CpG_MPs were enriched in 59 processes (P value < 0.01, S3 Table). Most of them were related to metabolic process. It is also noted that the 2,013 specific genes exclusively found by swDMR were enriched in 176 processes (P value < 0.01, S4 Table). Moreover, we found several processes related to differentiation were reappearing, including anatomical structure development, organ morphogenesis, multicellular organismal process, system development and embryonic organ morphogenesis, et al.

### Multiple samples

Methylome of hESC, hESC-Fibro and Firbo were applied to DMR detection for multiple samples.

When DMR detection was accomplished, we obtained 92,868 and 60,976 DMRs for swDMR and CpG_MPs respectively (S1 Table). There are 65.88% (40,169 in 60,976) DMRs of CpG_MPs overlapped with that of swDMR, and related to 1,473 genes which were annotated by DMRs (Fig 3D and 3E).

Then, enrichment analysis of biology process was performed with the same procedures used for two samples. Two hundred and fifteen significantly enriched biology processes (P value < 0.01, S5 Table) were obtained with same method of two samples DMR detection. Amounts of development related processes were enriched, such as multicellular organismal process, system development, cell motility, cell differentiation, embryonic appendage morphogenesis, skeletal system development. Of the specific 421 genes of CpG_MPs, 15 biology processes were identified (P value < 0.01, S6 Table). Of the specific 2,641 genes of ANOVA, 185 biology processes were enriched (P value < 0.01, S7 Table). Multicellular organismal process, anatomical structure morphogenesis, developmental process and embryonic organ development were also identified. Among those 2,641 specific genes, methylation of KLF4 promoter increases with the differentiation process. swDMR identified longer region than that of CpG_MPs. In addition, swDMR also identifies longer region in FOXO4 promoter (Fig 3F). Lower methylation in promoter of FOXO4 in Fibro may be active as a negative regulator of cell cycle and involve in growth and differentiation.

In DMR detection of either two or three samples, swDMR find more genes related to development from hESC to hESC-Fibro as well as hESC-Fibro to Fibro. It provided abundant information to comprehend the DNA methylome of differentiation process.

### DMR annotation

**Fig 4:**
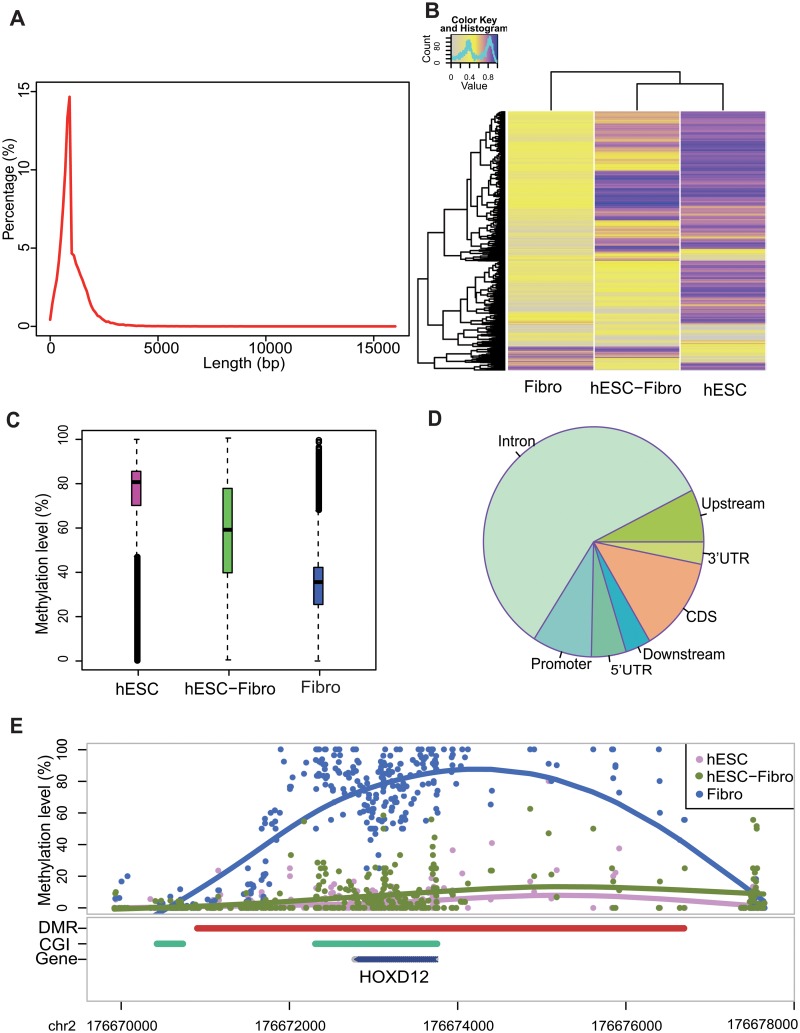
Results of swDMR. (A) Length distribution of DMR. (B). Methylation level cluster analysis of three samples (hESC, hESC-Fibro, Firbo). (C) Methylation level boxplot of three samples. (D) Enrichment analysis of genome features (5-UTR, 3-UTR, CDS, Intron, Upstream, Downstream). (E) A specific DMR related to HOXD12 gene.

swDMR also provides other useful tools to further comprehensively annotate DMRs, such as length distribution of DMRs, cluster analysis and visualization. Take DMR detection of three cell lines with ANOVA in swDMR for example. Statistical analysis of length distribution showed that DMRs are distributed especially numerous in 1000bp (Fig 4A). Through clustering analysis, complete linkage hierarchical clustering of the methylation level for each C in the CG sequence context was acquired (Fig 4B). The similiarity between hESC and hESC-Fibro is higher than that between hESC-Fibro and Fibro. The boxplot of methylation level of DMRs could be acquired (Fig 4C). It also suggests the decreasing trend of DNA methylation level from hESC, hESC-Fibro to Fibro in DMRs, consistent with the whole genome methylome change during differentiation[12]. Subsequently, all DMRs were mapped to genomic features and distribution of DMRs in different genomic features were obtained through enrichment analysis (Fig 4D). And a significant DMR covered the whole HOXD12 gene related to developmental regulation (Fig 4E). 21 DMRs were identified in the region around HOXD family genes and visualized on UCSC genome browser with default track option and CpG island option (S1 Fig).

### Performance

swDMR is well balanced with speed and memory consumption that can identify DMRs across samples from the large dataset of WGBS efficiently. It implements Perl and R language, and can run in parallel to accelerate the process of DMRs detection. Here, we performed swDMR with whole genome bisulfite sequencing data on high-performance computer with Red Hat 4.1.2–48 operate system, AMD CPU (2.2GHz) with multiple CPUs. It took about 6 hours to finish DMR detection on 10 threads. In terms of memory consumption, it costs less than 30 Mb memories in DMR detection, annotation and visualization, and about 260 Mb memories in clustering analysis.

## Perspectives

Our main objective in developing swDMR is to provide comprehensive survey of DMRs detection from WGBS data. swDMR is capable of identifying DMRs and conducting comparison analysis based on methylation profiles of two or multiple samples without replicates. In addition, versatile statistical methods have been integrated into swDMR to suffice individual analytical demands. Currently, swDMR only provides paralleled DMR cluster analysis, annotation and visualization of DMRs. To better uncover the role of DMRs in epigenetic regulation and the potential function regions related to gene transcriptional regulation, we expect continuous efforts will be made to improve swDMR. In the future, swDMR will implement some databases and existing toolkits, such as KEGG[31], WEGO[32] and DAVID[33] for gene ontology and pathway enrichment analysis. Furthermore, swDMR will take into consideration of additional factors (transcription factors, histone modifications and other functional elements et al.) that can cooperate with DNA methylation to regulate transcriptional. Additionally, we will apply ENCODE data[29, 34] to investigate the potential functions and mechanisms of DMRs in human functional elements. Extended identification of imprinted loci with allele-specific DNA methylation (ASM) will be developed soon, and is expected to greatly facilitate epigenetics studies of complex disease[35]. In conclusion, swDMR is a robust software under active development that can flexibly and precisely identify DMRs from WGBS data, and lay a solid foundation for further functional genomics analysis.

## Supporting Information

S1 Fig WIG visualization. The WIG format file produced by swDMR was visualized on UCSC genome browser. 21 DMRs were displayed across regeion of HOXD family genes.
(EPS)

S1 Table DMR of two or three samples identified by fisher exact test, ANOVA of swDMR and CpG_MPs and DMR distribution of different gene features. (XLS)

S2 Table Biology process enrichment of swDMR and CpG_MPs shared genes with DMR located in promoter in two samples DMR detection. (XLS)

S3 Table Biology process enrichment of CpG_MPs specific genes with DMR located in promoter in two samples DMR detection. (XLS)

S4 Table Biology process enrichment of swDMR specific genes with DMR located in promoter in two samples DMR detection. (XLS)

S5 Table Biology process enrichment of swDMR and CpG_MPs shared genes with DMR located in promoter in three samples DMR detection. (XLS)

S6 Table Biology process enrichment of CpG_MPs specific genes with DMR located in promoter in three samples DMR detection. (XLS)

S7 Table Biology process enrichment of swDMR specific genes with DMR located in promoter in three samples DMR detection. (XLS)
